# Kooperation zwischen Kinder- und Jugendpsychiatrie und Kinder- und Jugendhilfe mit speziellem Schwerpunkt auf die Unterbringung von Kindern und Jugendlichen in sozialpädagogischen Wohnformen

**DOI:** 10.1007/s40211-020-00378-2

**Published:** 2020-11-26

**Authors:** Judith Noske, Leonhard Thun-Hohenstein

**Affiliations:** 1 Abteilung für Kinder- und Jugendpsychiatrie Hinterbrühl, Krankenhaus Mödling, Fürstenweg 8, 2371 Hinterbrühl, Österreich; 2 Universitätsklinik für Kinder- und Jugendpsychiatrie, Salzburger Landeskliniken, Campus Christian Doppler Klinik, Paracelsus Medizinische Privatuniversität, Ignaz Harrerstraße 75, 5020 Salzburg, Österreich; 3 Arbeitsgruppe Versorgung der Österreichischen Gesellschaft für Kinder und Jugendpsychiatrie, Psychosomatik und Psychotherapie (ÖGKJP), Wien, Österreich

**Keywords:** Kooperation, Liaison, Wohnunterbringung durch die Kinder- und Jugendhilfe, Cooperation, Liaison, Residential care, Child and youth wellfare

## Abstract

Kinder- und Jugendpsychiatrie und Kinder- und Jugendhilfe betreuen viele gemeinsame Kinder und Jugendliche. Insbesondere bei den schwierigsten Kindern und Jugendlichen ist eine Kooperation am meisten gefordert. Viele dieser schwierigen Kinder und Jugendlichen kommen aus schwierigen Lebensverhältnissen, sind häufig traumatisiert und zeigen stark externalisierendes Verhalten. Die Unterbringung in einer extrafamilialen Wohnform stellt die Beteiligten aller Systeme häufig vor große Probleme.

Die Kinder- und Jugendpsychiatrie kann auf allen Ebenen wertvolle Beiträge leisten. Einerseits im Rahmen der spezifischen Kinder- und Jugendpsychiatrischen Diagnostik und Behandlung, ganz besonders im Konsiliar- und Liäsondienst und/oder in kooperativen Einrichtungen oder Betreuungsformaten. Dieses Angebot wird anhand der von der Kinder- und Jugendhilfe angebotenen Wohnformen diskutiert und konkrete Vorschläge zur Umsetzung diese Kooperation dargestellt.

## Summary

Departments of child and adolescent psychiatry and child and youth welfare care for many children and adolescents jointly. Cooperation is particularly important for treating most difficult children and young people. Many of these children and adolescents face difficult living conditions, are often traumatized and exhibit strongly externalising behaviour. Residential extra familial care settings often pose major problems for all professional partners. Departments of child and adolescent psychiatry can make valuable contributions at all levels. First, concerning specific child and adolescent psychiatric diagnosis and treatment, especially in consultant and liaison service and second supporting cooperative institutional care formats. A concrete proposal for a possible cooperation between departments of child and adolescent psychiatry and child and youth welfare care are discussed.

## Hintergrund

Das medizinische Sonderfach Kinder- und Jugendpsychiatrie (KJP) und die Kinder- und Jugendhilfe (KJH) sind zwei sehr unterschiedliche Systeme, die sich mit unterschiedlichem Aufträgen um beinahe dieselbe Gruppe von Menschen kümmern [1]. Beide Systeme haben das Kindeswohl im Fokus und bemühen sich zu einer Verbesserung der Lebenssituation von benachteiligten und/oder psychisch kranken Kindern und Jugendlichen beizutragen. Beide Fachbereiche wissen um die Wichtigkeit einer guten Kooperation. Diese bedarf des gegenseitigen Bemühens um Verständnis und Abstimmung sowie eine fortwährende dialogische Klärung der unterschiedlichen Aufträge. Jungmann [2] formuliert im Handbuch Jugendhilfe-Jugendpsychiatrie, dass das Konstrukt der Kooperation darauf abzielt, interdisziplinäres Handeln – mit dem Ziel einer vernetzten Problemlösung – zu erreichen. Mittel dazu ist das kreative Zusammenführen der vorhandenen Leistungsstrukturen und dies inhaltlich auszugestalten und beständig weiterzuentwickeln.

Insbesondere ist diese Zusammenarbeit gefragt, wenn es um massive Lebensveränderungen der Kinder und Jugendlichen geht, wie es z. B. eine Fremdunterbringung in einer staatlichen oder privaten Einrichtung darstellt. Von einigen Sozialpädagog*innen [3] wird die Fremdunterbringung als ein potenziell kritisches Lebensereignis angesehen. Dabei handelt es sich um Ereignisse, die Menschen aus ihrer Lebensbahn werfen, indem sie ihr alltägliches Leben unterbrechen und biographisch wirksam sind. Das „Kritische“ daran ist, dass das bisherige Welterlebnis basal verändert und persönliche Werte und Ziele dabei in Frage gestellt werden. Blandow [4] empfiehlt daher drei wesentliche Gesichtspunkte zu berücksichtigen: die Einbeziehung der Kinder und Jugendlichen in den Prozess der Fremdunterbringung, eine gute Organisationsstruktur der Einrichtung und ausreichende und ausreichend qualifizierte Betreuer*innen. Damit könnten die Ziele einer gelingenden Unterbringung, einer qualitativ guten Entwicklungsförderung erreicht und Abbrüche vermieden werden.

Ziel dieses Artikels ist es diese letzten Punkte um die Einbeziehung der KJP zu erweitern und einen Beitrag zum gegenseitigen Verständnis zu leisten. Weiters soll im Bereich der Fremdunterbringung/Wohnversorgung psychisch kranker Kinder und Jugendlicher eine fachlich-konzeptuelle Weiterentwicklung angeregt werden.

Kooperation als gesetzlicher Auftrag ist als solches nicht verankert und daher von individuellen und regionalen Personen und Prozessen sowie den – zumeist nicht – vorhandenen Ressourcen der beteiligten Systeme abhängig. Das Leitprinzip der Kooperation zwischen KJP und KJH ist die Übernahme einer gemeinsamen Verantwortung für die gemeinsame Klientel bei gleichzeitiger Anerkennung der fachlichen Autonomie des jeweils anderen und ein damit verbundener kollegialer Dialog. Im Zentrum dieses gesetzlichen Auftrages steht das „Kindeswohl“, wie es vom Gesetzgeber formuliert wurde (§138 STGB). Aus dieser gemeinsamen Verantwortung heraus und der Anerkennung der jeweiligen Autonomie des anderen entsteht eine verbindliche Kooperation mit Verständigung und Abstimmung der jeweiligen spezialisierten Arbeit unter Einbeziehung der Kinder und Jugendlichen und deren Familien. Bei multipel (auf sozialer und psychischer Ebene) belasteten Kindern und Jugendlichen braucht es hier ein übergeordnetes „interinstitutionelles“ Containment, das durch kooperative Konzepte zwischen einzelnen Trägern und entsprechenden Ressourcen auf Planungsebene gestützt und von der Politik abgesichert ist.

Für eine gesunde Entwicklung braucht es neben der Familie weitere Unterstützungssysteme (Kindergärten, Schulen, Nachbarn, Therapeut*innen etc.), die als „Brückenbauer“ fungieren: d. h. die zwischen den intrapsychischen und interpersonalen Schwierigkeiten vermitteln, die sich um Austausch und Vernetzung zwischen den, für die Entwicklung dieser Kinder wichtigen, Lebensbereichen bemühen.

Bei hoher Ausprägung des expansiven Verhaltens, bei chronischer Selbst- und Fremdaggression, sowie bei strukturellen Engpässen führt dies zu einer Überforderung der Helfer*innen und macht die Grenzen der Systeme sichtbar. Kinder und Jugendliche als „Systemsprenger“ [5] werden dann zu einer besonderen Herausforderung in der Kooperation zwischen KJH und dem Gesundheitssystem. Diese Überforderung einzelner Systeme führt zu „Verschiebepraktiken“ [6] und einer damit verbundenen Chronifizierungsgefahr. Andere Kinder gehen für die Systeme und sich verloren [7].

Viele Kinder und Jugendliche, die in Kinder- und Jugendhilfeeinrichtungen untergebracht sind, leiden an einer psychiatrischen Erkrankung und viele Familien mit psychisch kranken Kindern brauchen Unterstützung durch die KJH. Müller-Luzi [8] konnten in ihrer Studie zeigen, dass nach Einschätzung der pädagogischen Mitarbeiter*innen in Wohngemeinschaften 71,6 ± 22,8 % der Kinder und Jugendlichen psychische Auffälligkeiten zeigten. Hingegen wurden nach einer Studie von Beck [9] bei 46,5 % der Kinder und Jugendlichen nach einer stationären kinder- und jugendpsychiatrischen Behandlung eine stationäre Jugendhilfemaßnahme empfohlen. Aus diesen beispielhaften Zahlen lässt sich unschwer der Bedarf einer Betreuung durch beide Systeme ableiten.

In Österreich waren 2019 7800 Kinder (88/1 Mio Einwohner*innen) und Jugendliche in der stationären Jugendhilfe untergebracht und 12.780 (96/1 Mio Einwohner*innen) waren in voller Erziehung (Statistik Austria 2020). In Tab. 1 sind diese Daten aus dem Jahr 2016 für alle Bundesländer dargestellt und zeigen, dass es beträchtliche regionale Unterschiede gibt. Im Vergleich zeigt sich auch, dass 2019 etwas weniger Kinder und Jugendliche in stationären Jugendhilfeeinrichtungen untergebracht wurden [10].

**Table 1:** 

	EW (2016)	Soz.pädag. Einrichtung	SPE/Mio EW	Volle Erziehung	VE/Mio EW
Wien	1.840.000	2217	120,49	3921	213,10
NÖ	1.654.000	1271	76,84	1969	119,04
OÖ	1.454.000	1121	77,10	1861	127,99
SBG	545.815	589	107,91	831	152,25
Tirol	739.139	609	82,39	845	114,32
Vlbg	384.147	323	84,08	587	152,81
Ktn	560.482	828	147,73	1122	200,18
Stmk	1.232.000	1181	95,86	2100	170,45
Bgld	291.011	284	97,59	410	140,89
Ö Gesamt	8.700.594	8423	96,81	13.646	154,56
Ö 2019	8.859.000	7800	88,05	12.785	144,32

**Figure 1:**
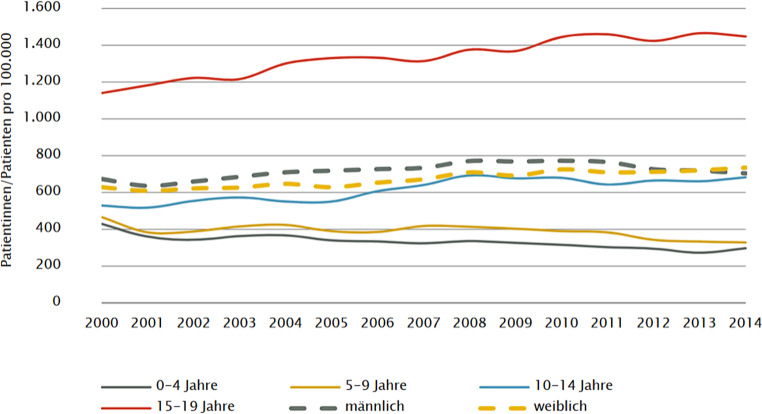

Die Häufigkeit stationärer Aufnahmen an Kinder- und Jugendpsychiatrischen Abteilungen lassen sich aus der Diagnosen- und Leistungsdokumentation der Krankenhäuser erhalten und Abb. 1 zeigt die Daten der Gesundheit Österreich GmbH [11] im Kinder und Jugendgesundheitsbericht 2016. Insgesamt waren 2014 – der jüngste Bericht aus 2019 liegt noch nicht vor – ca. 2700 Kinder und Jugendliche auf 100.000 Einwohner stationär an einer KJP aufgenommen, wobei hervorzuheben ist, dass bei >15-Jährigen die höchste Aufnahmefrequenz zu beobachten war (1400/100.000 Einwohner*innen).

### Kinder- und Jugendpsychiatrische Erkrankungen

Fachlicher Hintergrund der Notwendigkeit einer Kooperation sind die zugrundeliegenden psychiatrischen Erkrankungen. Eine psychische oder seelische Störung ist ein Zustandsbild, das durch krankheitswertige Veränderungen des Erlebens und Verhaltens gekennzeichnet ist. Psychische Störungen sind typischerweise mit deutlichem persönlichem Leidensdruck oder Belastungen und Problemen in mehreren Lebensbereichen verbunden [12]. Ca. 24 % aller Jugendlichen in Österreich leiden aktuell an einer psychischen Erkrankung, über ein Drittel aller Jugendlichen zeigt über den Verlauf ihres Lebens Symptome einer seelischen Erkrankung [13].

Zur Diagnosestellung von psychiatrischen Erkrankungen im Kindes- und Jugendalter wird das Multiaxiale Klassifikationsschema (MAS, [14]) des ICD-10 verwendet. Dieses Klassifikationsschema versucht neben der kinder- und jugendpsychiatrischen Diagnose nach ICD-10 auch psychosoziale Anpassung, Begabungsniveau, körperliche Erkrankungen sowie psychosoziale Belastungen zu erfassen und darzustellen. Insofern ist dieses Klassifikationsschema von erheblichem Informationsgehalt auch für andere Berufsgruppen.

Im Zentrum der Kinder- und Jugendpsychiatrischen Behandlung steht – entsprechend der multiaxialen Diagnostik – ein multimodaler Therapiezugang. Ausgehend von einem ganzheitlichen Menschenbild umfasst die kinder- und jugendpsychiatrische Behandlung in erster Linie kinder- und jugendpsychiatrische sowie psychotherapeutische Zugänge. Weiter gehören alle therapeutischen Angebote zur Verbesserung des Selbstverständnisses, der Selbstwahrnehmung und der Selbstwirksamkeit (alle funktionellen Therapien wie Ergotherapie, Physiotherapie, Logopädie, Teilleistungstraining, Musiktherapie, Tier-gestützte Therapie …) dazu.

Die meisten psychisch belasteten Kinder und Jugendlichen können ambulant behandelt werden. Bei Versagen ambulanter Möglichkeiten und komplexer Symptomatik kann eine geplante stationäre Aufnahme zur umfassenden kinder – und jugendpsychiatrischen Diagnostik oder ein zeitlich umgrenzter therapeutischer Aufenthalt bei klar umschriebenen und vereinbarten Zielen indiziert sein. Bei akuten Gefährdungsmomenten und Zusammenbruch des haltenden sozialen Systems wird eine kurzfristige stationäre Krisenintervention notwendig. Eine Unterscheidung zwischen psychosozialer bzw. pädagogischer und psychiatrischer Krise [15, 16] ist notwendig, um daraus die entsprechenden Konsequenzen abzuleiten. Das Krisenmanagement soll nach einem in Kooperation vereinbarten Stufenplan erfolgen. Während bei der psychosozialen/pädagogischen Krise der Situationsbezug nachvollziehbar ist und eine gewisse Steuerbarkeit vorhanden bleibt und die Deeskalation möglichst innerhalb des Systems erfolgen soll, ist bei einer psychiatrischen Krise die Nachvollziehbarkeit häufig nicht mehr gegeben. Die psychiatrische Krise verlangt eine kinder – und jugendpsychiatrische Abklärung mit entsprechenden Interventionen.

### Kinder- und Jugendpsychiatrische Behandlungsstrukturen

Die Kinder und Jugendpsychiatrie in Österreich ist ein junges Fach, das in laufender Entwicklung ist. Sie existiert im engeren Sinne erst seit 1975, als eigenständiges Sonderfach erst seit 2007 [17]. Die Versorgungslage (Details siehe auch Karwautz und Fliedl in diesem Heft) hat sich aufgrund der bundesweiten Vorgaben im ÖSG [18] im letzten Jahrzehnt wesentlich verbessert. Mittlerweile gibt es – bis auf das Burgenland – flächendeckend eine Akut- und Versorgungspsychiatrie in allen Bundesländern. In den letzten Jahren sind Rehabilitationszentren für mentale/geistige Gesundheit dazugekommen. Spezialabteilungen zu bestimmten Krankheitsbildern (Drogen, Essstörungen, Persönlichkeitsstörungen, Forensik …) für Langzeittherapieaufenthalte fehlen in Österreich. Alternativ stehen neben unterschiedlichen tagesklinischen Modellen (an die stat. Abteilung angeschlossen oder disloziert) tagesstrukturierende, tagesklinische, nachtklinische Angebote zur Verfügung.

Einen konzeptuell und strukturell verankerten Konsiliar- und Liaisondienst zu Einrichtungen der Pädiatrie und KJH gibt es nur punktuell (Arbeitsfortschrittsbericht zur integrierten psychosozialen Versorgung^1^), mittlerweile ist diese Kooperation im Österreichischen Strukturplan Gesundheit verankert [18].

### Wohnformen der Kinder- und Jugendhilfe

Die stationäre Jugendhilfe ist je nach Bundesland unterschiedlich strukturiert. Im Bundesland Salzburg gibt es beispielsweise, entsprechend der Salzburger KJH Wohnverordnung [19], folgende Einrichtungstypen: betreute Wohngemeinschaften für Kinder und Jugendliche (Gruppengröße 8 Bewohner*innen), ambulant betreutes Wohnen für Jugendliche (Gruppengröße 2), Kriseneinrichtungen für Kinder und Jugendliche (Gruppengröße 4) und intensiv betreutes Wohnen für bis zu 6 Kinder und Jugendliche Die NÖ Kinder- und Jugendhilfeeinrichtungs-Verordnung [20] definiert im seit 2020 wirksamen Normkostenmodell folgende Strukturen: Sozialpädagogisch inklusive Gruppen mit dem Modul Individualbetreuung, Familienähnliche Wohnformen, Krisenzentrum, Mutter-Kind-Wohnen, begleitete Verselbständigung und therapeutische/intensivpädagogische Kleinstgruppe. Es werden pädagogische und medizinische Voraussetzungen für die stationäre Unterbringung definiert, ebenso Personalqualifikationen und -schlüssel. Gruppengrößen werden wie folgt definiert: Sozialpädagogische Einrichtungen mit bis zu max. 9 minderjährige Personen je Gruppe, mit bis zu max. 8 minderjährige Personen je Gruppe, wenn eine minderjährige Person unter drei Jahren alt ist; Familienähnliche Wohnformen (mit max. 5 minderjährigen Personen); Krisenzentren: bis zu max. 9 minderjährige Personen je Gruppe und Mutter‑/Kind-Einrichtungen: mit max. 9 minderjährige Mütter je Gruppe. Die Stadt Wien – als größter Versorger – strukturiert die sozialpädagogischen Einrichtungen in Betreuungseinrichtungen für Notsituationen, Betreuungseinrichtungen für die dauerhafte Betreuung von Kindern und Jugendlichen, betreute Wohnformen für Jugendliche und als einziges Bundesland auch nicht ortsfeste Formen der Sozialpädagogik [21].

## Vorschlag einer kooperativen Annäherung an die stationäre Jugendhilfeunterbringung

Der Großteil der Kinder und Jugendlichen lebt bei zumindest einem Elternteil. Das heißt die prinzipielle Stoßrichtung der sozialen, finanziellen und therapeutischen Unterstützung muss in Richtung Unterstützung des Familiensystems gehen. Die Herausnahme aus dem familiären System darf, besonders wenn es mit Widerstand des Kindes und der Eltern verbunden ist, nur nach genauer Abwägung und nie leichtfertig geschehen.

Infolge sollen einige idealtypische – am pädagogisch/therapeutischen Unterstützungsbedarf orientierte Wohnformen diskutiert werden.

- Krisenzentrum
- Einzelintensivbetreuung zur vorübergehenden Krisenunterbringung
- Sozialpädagogische Wohngemeinschaften mit maximal 9 Plätzen
- Sozialtherapeutische Wohngemeinschaften mit maximal 6 Plätze
- Psychiatrische Intensivgruppe zumeist für sehr kleine Gruppen (z. B. <4)
- Übergangswohnungen im Übergang zum Erwachsenenalter (Transition).

In welcher Wohnform ein Kind möglichst gut betreut werden kann, hängt von unterschiedlichen Faktoren ab, die in einer ausführlichen psychosozialen Diagnostik eingeschätzt werden müssen. Neben sozialen, sozialpädagogischen und sozialarbeiterischen Kriterien oder gerichtlichen Aspekten sollte eine komplexe kinder – und jugendpsychiatrische sowie psychologische Diagnostik als Entscheidungsgrundlage herangezogen werden (siehe auch Tab. 3 und 4) [22], um die Schwere der psychosozialen und kinder- und jugendpsychiatrischen Beeinträchtigung einzuschätzen (Schwere des Störungsbildes, psychosoziales Funktionsniveau, Reflexionsgrad, soziale Kompetenz und Gruppenfähigkeit, …). Es gilt abzuwägen, welche strukturellen, therapeutischen und schulisch/beruflichen Unterstützungen notwendig sind, wie viele Personen gleichzeitig sozial überblickt werden können, mit welchen Krisen zu rechnen ist und wie diese durch ein ausreichendes Krisenmanagement zu deeskalieren sind. Unterschiedliche störungsspezifische Konzepte der Einrichtungen (Konzept zur Eingliederung ins Berufsleben, traumapädagogische und -therapeutische Konzepte, sozialintegrative Konzepte …), sind ebenfalls zu berücksichtigen.

Eine gezielte Zuweisung zur Wohneinrichtung ist wichtig, um eine optimale Passung zwischen den Bedürfnissen des Kindes und den Möglichkeiten der Einrichtung zu finden und so weitere traumatische Brüche durch Einrichtungswechsel in der Biografie dieser jungen Menschen zu vermeiden.

In der Empfehlung der geeigneten Betreuungsform müssen neben den spezifischen emotionalen Schwierigkeiten und Verhaltensauffälligkeiten des Kindes/Jugendlichen, Strukturen und Möglichkeiten der Einrichtung und psychiatrischer/therapeutischer/pädagogischer Behandlungs- und Betreuungsbedarf und Ausmaß an notwendiger Kooperationen zwischen den Helfersystemen, im Sinne einer integrativen Behandlung, berücksichtigt werden [4].

**Figure 2:**
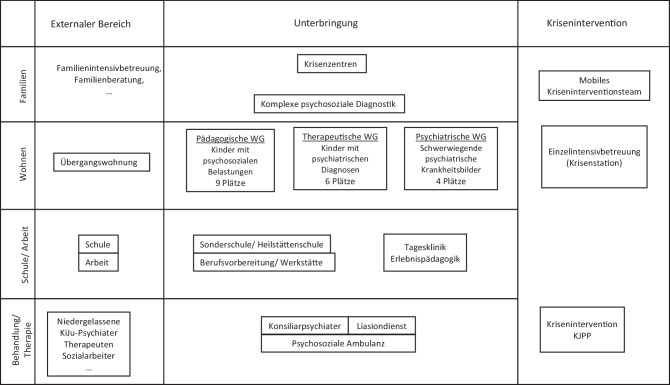

In Tab. 2 wurde der Versuch unternommen, die angeführten Unterbringungsformen nach Zielgruppe, Anzahl der Wohnplätze, nötigen Berufsgruppen, kinder- und jugendpsychiatrischen Beschreibung der Kinder und Jugendlichen und ihrer Psychopathologie darzustellen. Ebenfalls wird der mögliche Beitrag von Kinder- und Jugendpsychiatrie, Psychologie und Psychotherapie aufgeführt und die Wertigkeit des Ausbildungsthemas sowie die Zielsetzung der Versorgung. In Abb. 2 findet sich eine Übersicht der verschiedenen Angebote in Bezug auf die Verortung des Angebotes zwischen intra- und extramural.

**Table 2:** 

	Krisenzentrum	Sozialpädagogische WG	Therapeutische WG	K + J-psychiatrische Intensivgruppe	Übergangswohnungen im Übergang zum Erw.alter
Definition	Kurzfristige Möglichkeit der Unterbringung in Krisen	Langfristige, Entwicklungsbegleitende Unterbringung	Langfristige Entwicklungsbegleitende und therapeutische WG	Mittelfristige, psychiatrische therapeutische WG	Mittelfristige, sozialpädagogische WG
Wohnplätze	Bis zu 9	Bis zu 9	Bis zu 6	Bis zu 4	Offen
Qualifikation der MA	Sozialpädagoginnen, PsychologInnen, Krisenintervention	Sozialpädagog*innen; Traumapädagogisches Know-how	Sozialpädagog*innen; Therapieausbildung Traumapädagogisches Know-how; Kenntnisse psychiatrischer Erkrankungen und Umgang damit	Sozialpädagog*innen; Psycholog*innen, Pflege, Ergotherapie Traumapädagogisches Know-how; Kenntnisse psychiatrischer Erkrankungen und Fertigkeiten im Umgang damit; integratives psychiatrisches Konzept	Sozialpädagog*innen, Transitionskonzepte; Erfahrung mit Jugendlichen und Erwachsenen
Kinder +Jugendliche	K + J und System von akuter Belastung überfordert; unklare Gesundheitssituation	Psychosozial belastete, mehrheitlich gesunde K + J; gutes Strukturniveau	K + J haben bestätigte KJP-Diagnosen; KJP-Behandlung i. enger Kooperation mit WG; mittleres Strukturniveau	K + J haben schwerste K + J-psychiatrische Erkrankungen; schwerwiegende Defizite der Selbstregulation und Beziehungs- +Gruppenfähigkeit; geringes Strukturniveau	K + J haben bestätigte Erkrankungen
Psycho-pathologie	Im Rahmen der Krise	Posttraumatische/Trauma-bedingte Symptome; KJP-Störungen geringen Ausmaßes	Gesamtes Spektrum der KJP-Diagnosen	Gesamtes Spektrum der KJP-Diagnosen in extremer Ausprägung	Mittleres bis gutes Strukturniveau
Angebote der KJP-Versorgung	Krisenintervention Abklärung psychiatrischer Symptome	Abklärung psychiatrischer Symptome, KJP-Diagnostik und Behandlung	KJP-Diagnose und Behandlung; multimodale Versorgung, Kooperative Strukturen	Integratives Konzept über die Systeme hinweg	KJP-Diagnose und Behandlung; multimodale Versorgung, Kooperative Strukturen
Psychol-psychoth. Versorgung	Krisenintervention psycholog. Diagnostik	Psycholog. Diagnostik und Intervention; Psychotherapie	Psycholog. Diagnostik und Intervention; Psychotherapie	Integratives Konzept über die Systeme hinweg	Transitionskonzepte u. Einbeziehung d. Erwachsenen Psychiatrie
Ausbildung	Kein Thema	Zentrales Thema	Zentrales Thema	Entsprechend des jeweiligen Entwicklungs- und Strukturniveaus	Zentrales Thema inkl. Berufstätigkeit
Ziel	Rückführung ins Herkunftssystem; Entscheidung über weitere Versorgung	Erreichung der selbständigen Lebensfähigkeit	Selbststeuerungsfähigkeit, Beziehungsfähigkeit, Arbeitsfähigkeit, Erwerbsfähigkeit etc	Selbststeuerungsfähigkeit, Reduktion von Aggressivität Gruppenfähigkeit, Reduktion des abweichenden Verhaltens	Selbständigkeit Arbeitsfähigkeit

**Table 3:** 

Medizinisch-psychologische Diagnostik	
MAS	1. Klinisch psychiatrisches Syndrom 2. Umschriebene Entwicklungsstörungen 3. Intelligenzniveau 4. Körperliche Symptomatik 5. Assoziierte aktuelle abnorme psychosoziale Umstände 6. Globale Beurteilung des psychosozialen Funktionsniveaus
OPD-KJ‑2	Psychische Struktur Beziehungsverhalten Dysfunktionale Konflikte Behandlungsvoraussetzungen (Subjektive Krankheitshypothese, Leidensdruck, Veränderungsmotivation, Krankheitsgewinn, intrapsychische, familiäre und soziale Ressourcen, …)

*MAS* Multiaxiale Klassifikationsschema [14], *OPD-KJ* Operationalisierte Psychodiagnostik – Kinder und Jugendliche 2 [27]

**Table 4:** 

Diagnostische Einschätzung der Kinder und Jugendhilfe (vgl. Harnach Beck [29])	
Psychosoziale Diagnostik in der Kinder- und Jugendhilfe	Materielle Situation Zustand des Kindes Soziale Situation – Familiendiagnostik
Sozialpädagogische Einschätzung	Bindungsverhalten und Beziehungsgestaltung Beobachtungen im jeweiligen Lebensfeld Leistungsverhalten
*Lern- und Förderdiagnostik der Schule/Arbeitssituation*	
Schulischer und beruflicher Leistungsstand	Gegenwärtige Schulsituation Arbeitssituation und relevante Faktoren zur beruflichen Reintegration Schullaufbahn Diagnostische Beobachtungen in der Schule/Arbeitssituation

## Diskussion

Die innerpsychischen Probleme vieler Kinder und Jugendlicher stehen in engem Zusammenhang mit ihren Lebensbedingungen. Je problematischer die Lebensbedingungen sind, umso höher ist die Wahrscheinlichkeit auffälliges Verhalten, im weitesten Sinne, zu entwickeln. Interventionen der KJH zielen in der Regel darauf ab, die Lebensbedingungen zu verändern oder zu verbessern und durch gezielte Unterstützung ein lebenswertes Leben zu ermöglichen. Jedes Kind braucht einen sicheren Ort, ein Zuhause, es braucht Menschen, die es verstehen, es unterstützen und eine möglichst unbelastete Entwicklung gewährleisten. Die KJP versucht die Frage zu beantworten, ob ein krankhafter Zustand vorliegt und übernimmt die Verantwortung für die Behandlung. Aufgrund des multifaktoriellen Krankheitsverständnisses versteht sich die KJP-Betreuung als ein „wrap-around-service“ [4], d. h. sie fühlt sich nicht nur für die rein medizinischen Belange zuständig, sondern das Ziel der Behandlung ist die Lebensbewältigung an sich. Weiterentwicklungen der KJP können und müssen hier in Zukunft ebenfalls berücksichtigt werden: Home-treatment [23] und Integrative Versorgung [24]. Es handelt sich dabei um unterschiedliche, nachgehende und integrative Versorgungskonzepte, vor allem für schwer und schwerstkranke Kinder und Jugendliche. In der BRD wurden diese „stationsäquivalenten Leistungen“ als psychiatrische Behandlungsform in den Katalog der Krankenhausleistungen aufgenommen. In Österreich kämpfen wir noch um Pilotprojekte. Diese Erweiterungen der Kinder- und Jugendpsychiatrischen Versorgung können gerade bei Kindern und Jugendlichen in Einrichtungen der KJH ganz wesentliche Vorteile nach sich ziehen.

## Zusammenfassung und Aussicht

Die KJPP und die KJH sind zwei komplementäre Systeme, die aufgrund überschneidender Interessenslagen häufig in Konflikt und Konkurrenz stehen. Gemeinsame Interessen der beiden Systeme sind – neben dem Wohl der ihnen anvertrauten Personen – präventive Ansätze, dezentrale Versorgung, Alltagsorientierung, Integration und Partizipation. Im Rahmen der Hilfeplanung sollten daher die Kinder- und Jugendpsychiater*innen hinzugezogen werden, um durch den lebensgeschichtlich orientierten, multimodalen Zugang die Hilfeplanung anzureichern. In der BRD ist dies im § 35a des Kinder- und Jugendhilfegesetzes festgeschrieben [25]. Hilfreich sind dabei unter anderem die Einschätzung der Behandlungsvoraussetzungen, die Erhebung der subjektiven Krankheitstheorie und die Diagnostik der Persönlichkeitsstruktur (vgl. OPD-KJ‑2, [26]). Diese Einschätzung, die sich an der subjektiven Wahrnehmung und dem emotionalen Erleben des jeweiligen Kindes orientiert, kann die Indikationsstellung für eine entsprechende Wohnversorgung und von Begleitmaßnahmen sehr gut unterstützen.

Die Kooperation der beiden Systeme – KJH und KJP – hat für die betreuten Kinder und Jugendlichen erhebliche Bedeutung und kann damit die Wirksamkeit der verschiedenen Maßnahmen und den Outcome für die betreuten Kinder und Jugendlichen deutlich verbessern. Im ÖSG 2017 [18] sind verschiedene Grundsätze und Versorgungsvorschläge formuliert, die dem Anliegen dieses Artikels entsprechen, allerdings der Umsetzung harren. Neben einigen Grundhaltungen wie Partizipation, dezentrale Versorgung, Integration in die Primärversorgung wird vom Gesetzgeber festgehalten, dass es um den Aufbau regionaler sozialpsychiatrischer Netzwerke bzw. kinder- und jugendpsychiatrischer Netzwerke unter Einbeziehung aller Anbieterstrukturen (inkl. Sozial- und Behindertenbereich, Kinder- und Jugendhilfe) geht und dafür eine landesweite Netzwerkkoordination aufgebaut werden soll.

## Abkürzungen

KJP: Kinder- und Jugendpsychiatrie
KJH: Kinder und Jugendhilfe

## Footnotes

1 Steinberger, K. (2012). Sozialpsychiatrische Liaisondienste zu Einrichtungen. Unveröffentlichte Präsentation im Rahmen des Workshops „Psychosoziale Planungs- und Versorgungskonzepte für Kinder und Jugendliche“ Persönliche Mitteilung.
